# Comprehensive Aspectual UML Approach to Support AspectJ

**DOI:** 10.1155/2014/327808

**Published:** 2014-07-21

**Authors:** Aws Magableh, Zarina Shukur, Noorazean Mohd. Ali

**Affiliations:** Faculty of Computer Science and Information Technology, UKM, 43600 Bangi, Selangor, Malaysia

## Abstract

Unified Modeling Language is the most popular and widely used Object-Oriented modelling language in the IT industry. This study focuses on investigating the ability to expand UML to some extent to model crosscutting concerns (Aspects) to support AspectJ. Through a comprehensive literature review, we identify and extensively examine all the available Aspect-Oriented UML modelling approaches and find that the existing Aspect-Oriented Design Modelling approaches using UML cannot be considered to provide a framework for a comprehensive Aspectual UML modelling approach and also that there is a lack of adequate Aspect-Oriented tool support. This study also proposes a set of Aspectual UML semantic rules and attempts to generate AspectJ pseudocode from UML diagrams. The proposed Aspectual UML modelling approach is formally evaluated using a focus group to test six hypotheses regarding performance; a “good design” criteria-based evaluation to assess the quality of the design; and an AspectJ-based evaluation as a reference measurement-based evaluation. The results of the focus group evaluation confirm all the hypotheses put forward regarding the proposed approach. The proposed approach provides a comprehensive set of Aspectual UML structural and behavioral diagrams, which are designed and implemented based on a comprehensive and detailed set of AspectJ programming constructs.

## 1. Introduction

Object Orientation has the potential to enhance software modularity and reusability by using polymorphism and inheritance mechanisms. However, it lacks the ability to address the Separation of Concerns (SoC) concept [1]. Concerns are represented as Classes and Objects when analysing, designing, and programming Object Orientation. However, complex systems usually have “crosscutting concerns” which, as the term implies, cut across functional modules and components, thereby increasing their interdependencies, which leads to a reduction in their modularity and the generation of scattered and tangled code in the implementation solution. In the Object Orientation paradigm, these crosscutting concerns, which are also known as Aspects, are not efficiently represented in Object Orientation.

The complexity and size of systems have grown substantially in recent years. This has led to the manifestation of new concerns. These new concerns crosscut other concerns and core Classes in the system. The implementation solution for these crosscutting concerns is scattered throughout the components of the system, which results in the problem of code scattering and tangling. Code scattering and tangling make the components of the system hard to reuse, comprehend, and maintain. These drawbacks have led to the development and adoption of Aspect-Oriented (AO) concepts to handle the crosscutting concerns in all stages of the Software Development Life Cycle (SDLC). This approach is described as Aspect-Oriented Software Development (AOSD) [2]. As a result, the concept of SoC has been enhanced to generate a new concept known as Advanced Separation of Concerns (ASoC) [3]. However, the development of an improved approach to model and represent these crosscutting concerns is still critical to reduce or eliminate tangled code.

Advanced Separation of Concerns tends to separate crosscutting concerns such as logging and security from the core concerns of the system by using Aspect-Oriented Programming (AOP). It provides the implementation solution for the code scattering and tangling issues as a part of AOSD. Aspect-Oriented Programming overcomes the problem of code spreading over the core concerns—the issue encountered in Object Orientation when it implements the crosscutting concerns—by using a new implementation modularity unit called the “Aspect.” AspectJ is a type of AOP that has grown in popularity in the industrial environment and in academic research [4, 5], which has consequently increased the interest in investigating Aspect Orientation for other stages of the SDLC.

Since the modelling stage is critical, Aspect-Oriented Modelling (AOM) has become a hot topic in research circles. Aspect-Oriented Modelling focuses on the process of identifying, analysing, managing, and representing crosscutting concerns in AOSD. At the same time, the UML is the most well-known and widely used modelling language in the industry, so it is not surprising that there is also interest in investigating UML support for AOM.

## 2. Literature Review of the State of the Art in Aspect-Oriented Design Modelling

The main topics with which one has to be familiar in this field include UML [4, 5], UML extensions [6], Aspect-Oriented Design Modelling (AODM) [7], AOP, and AspectJ language constructs [8].

The researcher therefore conducted a systematic literature review (SLR) which focused on identifying, appraising, selecting, and synthesizing all the available high-quality research on state-of-the-art approaches on AODM that use UML. The SLR is a process that consists of reviewing, extracting, and evaluating and then analysing and interpreting the studies that are relevant to the three research questions of this research. (RQ1) How do the existing approaches that model Aspects (crosscutting concerns) use UML? (RQ2) What are the objectives of having a comprehensive approach that extends UML to support AOM in the early stage of AOSD? (RQ3) How beneficial is it to have a comprehensive approach that extends UML to support AOM based on AspectJ in the early stage of AOSD? Most research starts with a literature review. However, unless a literature review is thorough and fair, it is of little scientific value [9]. Hence, this review aims to synthesize existing work in a manner that is not only fair but seen to be fair.

The researcher identified 468 articles and books that are related to the research questions posed in this study. The researcher extracted all the directly related studies by removing duplication and redundancies and by reading the abstract and the full text of the articles. As a result, the researcher found 73 primary studies related to the topic investigated in this research. A breakdown of the study counts for the research questions at each stage of the screening process is shown in Table 1.

**Figure 1:**
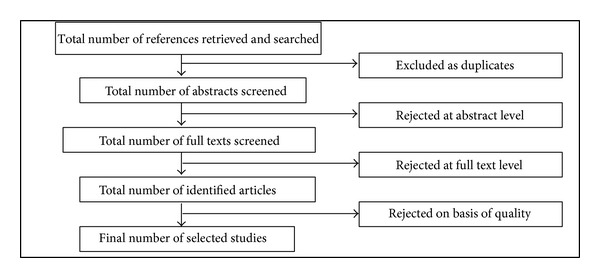
Flowchart of literature screening and selection process.

The screening process consisted of four steps; the flow of the steps is illustrated in Figure 1.

### 2.1. Existing AOM Approaches Using UML to Support AspectJ

Based on an analysis of the relevant studies, the researcher found that software analysis and design can be divided into Object-Oriented (OO) [10], Subject-Oriented [11], Feature-Oriented [12], and AO approaches [13]. Aspect-Oriented approaches can be further subdivided into Aspect-Oriented Requirement Engineering [14], AO Architecture [15, 16], and AODM. The latter (AODM) specifies the behaviour and structure of the software system [13].

This research work focuses on AODM and investigates how to represent and capture the dynamic and static view of crosscutting concerns in the early stage of the SDLC. Many research studies have developed different kinds of modelling approaches such as Theme/UML [17], Subject-Oriented programming [2], CoCompose [18], Aspect at Design Time (ADT) [19], and AO UML diagram modelling. The latter (AO UML diagram modelling) is the concern of this research.

The AO UML modelling approaches can be classified into two general categories. The first category consists of approaches that construct/build UML profiles. The construction of a UML profile extension is usually called a lightweight extension because it does not involve the implementation of any new UML metamodel elements. The UML profile extension approaches utilize UML constraints, tagged values, and stereotypes to model different domains. Thus the UML profile extension technique depends on the flexibility and extendibility of the standard UML [20]. The second category consists of approaches that extend the UML metamodel, which are considered to be heavyweight extensions because they involve developing a new UML metamodel to represent Aspects and their crosscutting nature [21].

By conducting an SLR following the above detailed steps, the researcher found that there are 14 relevant and main studies relevant to the topic addressed in this paper. Based on an analysis of these 14 studies, the researcher also categorized the existing UML modelling approaches into the following four types:

1. heavyweight UML: [22–28],
2. lightweight UML: [29–33],
3. mixed-mode UML (i.e., the combination of heavyweight and lightweight UML extensions): [34],
4. standard UML: [35].

### 2.2. Criteria Adopted for Comparison of AOM Approaches

**Figure 2:**
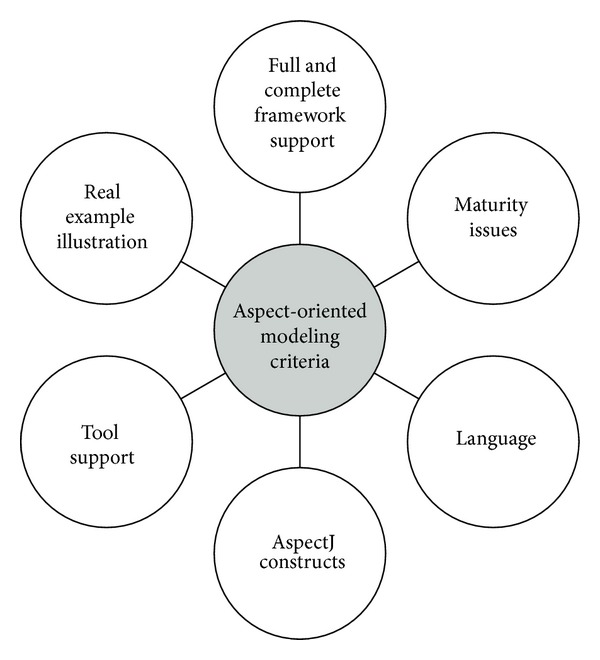
Criteria used to compare AOM approaches.

This section describes the six criteria used to compare the AO UML modelling approaches as well as their respective subcriteria. The six key criteria are illustrated in Figure 2. The motivation behind the selection of these criteria was to reveal the knowledge gaps and identify and show how this study aimed to fill those gaps. In other words, identification of these criteria provided a basis for a comprehensive analysis of existing AO UML approaches, which allowed comparison of different AO UML approaches in greater detail. A brief description of each criterion is given below.

#### 2.2.1. Language Specification (LS)

The LS comprises the following subcriteria that are related to the specifications of the modelling language: (i) the UML Modelling Language Version (UMLMLV), that is, the edition of UML that has been used; (ii) the Extension Mechanism (EM), that is, whether it is a UML profile extension (lightweight) or a UML metamodel extension (heavyweight); (iii) Language Purpose (LP), that is, whether an approach is a general or a specific domain model; (iv) Platform Dependency (PD), that is, whether an approach is specific to a selected platform or is suitable for cross-platform use; (v) Diagram Type (DT), that is, which types of UML diagram, structural and/or behavioural diagrams, are utilized by an approach; (vi) Modelling Process (MP) [36], that is, whether an approach has clearly stated processes for the modelling steps; (vii) Traceability (T), that is, whether the approach has the ability to track and trace the concern from the early stage of the software life cycle to the later stage; and (viii) Adaptability (A), that is, whether the approach has the capability of being extended to model a complex system.

#### 2.2.2. AspectJ Constructs (AJC)

AspectJ constructs contain vital subcriteria. All the AspectJ constructs are studied in a reverse engineering (bottom-up) approach to develop a modelling notation for all the AspectJ syntax that needs to be modelled and used in the early stage of software development. The aim is to increase the consistency and level of model understanding [37]. The criteria inspired by [38] are as follows: Dynamic Crosscutting Support (DCS), Static Crosscutting Support (SCS), Join Point Types (JPT), Pointcut Types (PCT), and Advice Types (AT).

#### 2.2.3. Maturity Issues (MI)

The term Maturity Issues (MI) relates to the maturity of the proposed approaches. As outlined above, many approaches have been proposed, but this study focuses on those that are well established and that have gained a good reputation among researchers [36]. The subcriteria of MI are divided into Modelling Examples (ME), Application in Real-world Projects (ARWP), and Usability (U).

#### 2.2.4. Tool Support (TS)

Tool Support (TS) is self-explanatory and has the following subcriteria: Modelling Support (MS), Code Generation (CG), which means generating AspectJ pseudocode, and Model Kernel Extraction (MKE), which refers to extracting and saving the model kernel into a portable file format such as Scalable Vector Graphics (SVG) XML.

#### 2.2.5. Real Example Illustration (REI)

The Real Example Illustration (REI) takes into consideration the applicability and reality of the demonstrated example used in the approach. The REI could be a Used Example (UE), which indicates that the example has been used before by other approaches. It could also have Complexity (C), which indicates that example is complex rather than trivial.

#### 2.2.6. Comprehensive Framework Support (CFS)

The term Comprehensive Framework Support (CFS) refers to the completeness of the existing approach. If an AO UML approach has CFS, this means that the approach represents Aspects in all UML diagrams.

### 2.3. Results of Comparison of Existing Approaches

As mentioned above, based on the results of the SLR, a total of 14 AO modelling approaches are relevant to the research questions. In this part of the study, these approaches are compared against the selected criteria briefly identified in Section 2.2. The discussion in this section is presented in a tabular format with explanatory text. The approaches are given in the rows and the criteria in the columns of the table. For ease of reference, a coding index that describes the meaning of each abbreviation is supplied at the bottom of the table. A separate table was created for each comparison criterion, but due to space limitations we only explain the Language Specification criterion herein (Table 2).

Table 2 illustrates the comparison between the selected approaches based on this criterion and its subcriteria. One of the most interesting findings is that none of the proposed approaches used UML 2.4 and none of them focus on all the UML diagrams; rather, the majority focus on the Class Diagram (structural modelling).

## 3. Problem Statement

Software systems have become more complicated because their size has grown dramatically as new distributed system modules and system components have been developed and, in addition, system users have become more diverse. The existence of such complex systems has led to the manifestation of new crosscutting concerns (Aspects) and these new concerns crosscut other concerns and core Classes. Many modelling techniques have been proposed to model these huge systems. One of the most well-known techniques is Object Orientation.

Object-Oriented modelling has many advantages and benefits that have been outlined in the literature and its usability in modelling software systems has been proved [39]. However, OO modelling has weaknesses in terms of modelling Aspects for the following three reasons: (1) it attempts to model an Aspect by treating it as an Object, which is semantically incorrect; (2) it does not have the modelling notations and mechanism to capture the constructs of the Aspect; and (3) it does not maintain a high level of consistency between the various software life cycle stages [40]. To date, there is still no consensus in the AOM community on a standard set of extension modelling notations [21, 41], but the AOM community is motivated to investigate and find a solution for the above mentioned issues.

As stated above, UML is one of the most well-known OO modelling languages in the industry [42, 43]. It has been built to support the concepts of Object Orientation. Taking into consideration that Aspect Orientation is an extension to Object Orientation [44], it appears to be logical to investigate whether it would be possible to extend UML to support Aspect Orientation. Indeed, many approaches have been proposed in this regard. The majority of these existing approaches depend on a UML lightweight extension mechanism to support Aspect modelling. According to the UML 2.4 infrastructure, the profile mechanism (lightweight) is not a first-class extension mechanism, where it does not allow modification to the existing metamodel; rather, the intention of profiles is to give a straightforward mechanism for adapting an existing metamodel. However, the first-class extensibility is handled through metamodel extension, where there are no restrictions on what one is allowed to do with a metamodel: one can add and remove metaclasses and relationships as one finds it necessary. Of course, it is then possible to impose methodology restrictions that one is not allowed to modify the existing metamodels but only extend them [45].

Furthermore, this research attempts to propose an approach to model Aspects because there is a lack of standard design notation in AOM to design an AO program [46]. Aspects are responsible for producing spread and tangle code representation that is spread across and tangled throughout the various software stages; hence an approach that can model and represent these crosscutting concerns effectively is vital.

Through the SLR conducted for this thesis, the researcher found that there are several proposed approaches for AODM using UML. However, there is no uniform standard [7, 47]. The researcher also found that there are a lot of weaknesses and drawbacks in the existing Aspect modelling approaches. This research study will attempt to overcome these drawbacks, which are listed below.

- The modelling of crosscutting concerns from the early stages is not fully supported.
- Current approaches do not consider a complete and comprehensive Aspectual UML approach because few UML diagrams have been proposed to support Aspects.
- The majority of the existing approaches depend on a UML lightweight extension to support Aspect modelling, but the lightweight extension is not considered to be a language extension that is expressive enough to model Aspects [6].
- Existing approaches propose nonstandard forms of modelling notations and extensions.
- Less attention is given to UML modelling to support AspectJ's detailed constructs.
- There is a lack of tool support for Aspect modelling.
- The mapping of AspectJ's detailed constructs from the programming level to the design level is not fully supported.
- None of the reviewed existing AO approaches propose modelling steps that a developer can follow to save time and effort.
- None of the existing approaches provide semantic rules to maintain the correctness of the modelling.
- None of the existing approaches provide the ability to generate a portable files format out of the UML drawings.
- None of the existing approaches provide the ability to generate AspectJ pseudocode.

This study focuses on bottom-up technique to invent an approach for modelling Aspects using UML to support AspectJ. It starts from the well-established Aspect programming language level (AspectJ), which is currently a hot topic in the programming domain [48], and moves upward to the design level to generate AO UML (Aspectual UML) diagrams. There is a lack of Aspectual modelling notations to support AOP, and specifically AspectJ [49], so it is necessary to examine the coding constructs and try to implement AOM by using a representational notation at the modelling level. Through that process, the researcher can attempt to increase the level of encapsulation, not only at the coding level, but also at the design level. In addition, by taking this approach, the level of consistency is maintained at a high level because all the coding constructs are denoted early on, at the design stage.

## 4. Research Methodology

**Figure 3:**
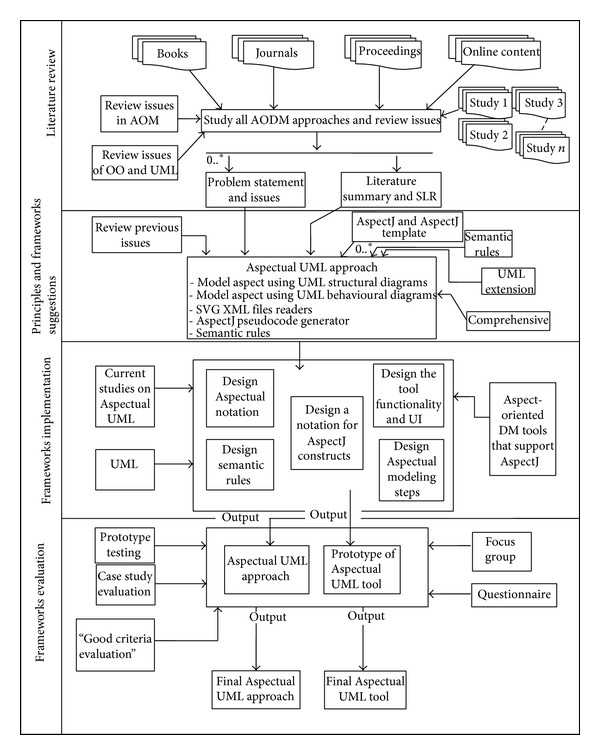
Conceptual framework.

This research study uses Design Science Methodology. The DSM is a set of synthetic and analytical techniques and perspectives that can be used when performing research. It involves the creation of new knowledge through the design of novel or innovative artefacts (things or processes that have or can have material existence) and analysis of the use and/or performance of such artefacts along with reflection and abstraction. These artefacts include, among others, algorithms (e.g., for information retrieval), human/computer interfaces, and system design methodologies or languages. Design science researchers work in many disciplines and fields, notably engineering and computer science, and they use a variety of approaches, methods, and techniques [50].  Figure 3 illustrates the problem statement within the context of a conceptual or theoretical framework. This type of description contributes to a research report in two key ways because it identifies the research variables and clarifies the relationships among those variables.

The general conceptual framework of this research consists of four phases. The first is the theoretical phase, which consists of a review of the literature (LR) on the relevant topics, issues, and problems of this research. The second is the principles and framework suggestion phase, which puts forward the proposed bottom-up methodology and the research framework. This includes AspectJ mapping from programming level to design and analysis level to model Aspects using Aspectual UML diagrams. In the third phase, the proposed methods and concepts are designed and implemented. In the fourth and final evaluation phase, several evaluations are performed including a focus group-based evaluation to validate the applicability of the proposed approach and tool. Also, the modularity of the proposed approach is evaluated using a “good design” criteria-based evaluation approach. The proposed concept is then evaluated by implementing and assessing a prototype tool that carries out the proposed theoretical part of this research work.

### 4.1. Phase 1: Theoretical Study

In this phase, many books, journals, online articles, and proceedings relevant to this research topic are reviewed. In this review, many Aspect-Oriented (AO) approaches and tools are screened and all Aspect-Oriented Design Modelling (AODM) approaches are reviewed. The main outputs of this phase are the problem statement and the literature summary. The outputs of this phase are considered as inputs to the next phase.

### 4.2. Phase 2: Principles Identification and Framework Development

In this phase, the principles and suggestions for a possible framework are consolidated in one place. After obtaining the problem statement and the literature summary, the issues found in the previous works are reviewed and consolidated. The initial process of developing the Aspectual UML approach starts in this phase. Different principles such as AspectJ support, completeness of the approach in terms of covering all Aspects, use of both types of UML extensions, and designing semantic rules to define the relationships between the AO and OO notations are investigated.

### 4.3. Phase 3: Framework Implementation

In this phase, the research moves to another level and the implementation of the conceptual framework identified in the previous phase starts. Designing the Aspectual UML modelling approach to support Aspects involves designing Aspectual notations that support Aspect constructs, designing the semantic rules between the notations, and designing the Aspectual modelling steps and the Aspectual UML (AUML) tool based on UMLet, which is an open-source UML tool. In this phase, AspectJ templates are reviewed in order to propose a method to generate AspectJ pseudocode.

### 4.4. Phase 4: Experiment and Evaluation

The Aspectual UML approach and the prototype AUML tool are implemented in this final phase. In this phase, both the Aspectual UML approach and the AUML tool are evaluated. Different kinds of evaluation techniques are used. To evaluate the proposed approach, three different qualitative methods are used: (1) a focus group-based evaluation is used to evaluate the applicability and correctness of the proposed approach, (2) a “good design” criteria-based evaluation is used to evaluate the modularity of the approach and (3) an AspectJ-based evaluation. Finally, the proposed tool is evaluated using black box and white box testing. These phases are elaborated as well in Table 3.

## 5. Aspectual UML Approach to Support AspectJ

The previous sections discussed the SLR and the research methodology. In that review, the researcher identified the current state of play with regards to research on Aspect-Oriented UML Design Modelling (AUDM) that supports AspectJ. Based on that review, the researcher proposes an extension to the published work by introducing an Aspectual UML modelling approach to support AspectJ. The proposed comprehensive approach introduces a new AUDM notation to model crosscutting concerns (Aspects) to support the AspectJ programming constructsmapped with AspectJ. It is intended to act as a complete reference guide to developers and researchers and it aims to provide an Aspectual Modelling Methodology (AMM) that can be followed in AOSD. The proposed AUDM notation is divided into two types: Aspectual UML structural diagrams that cover all UML structural diagrams, where a notation to represent AspectJ constructs is proposed for each diagram, and Aspectual UML behavioural diagrams that cover all UML behavioural diagrams, where a notation to represent AspectJ constructs is proposed for each diagram. This approach attempts to generate files in an XML SVG portable format which could help in the export of the drawings. Additionally, this approach attempts to generate AspectJ pseudocode out of the drawings. To support the proposed approach, a proof-of-concept prototype was implemented based on the UMLet open-source tool. The motivation for proposing a novel Aspectual UML to support the AspectJ approach can be found in the four intended main benefits.

1. It makes the implementation, coding, and development of AspectJ programs easier.
2. It makes use of the perception of AO concepts in design.
3. It helps programmers to evaluate the influences of the Aspects on the Classes.
4. It utilizes and facilitates the advantages of AO concepts such as adaptability and reusability.

### 5.1. Aspectual Modelling Methodology (AMM)

In this section, the researcher presents the proposed AMM, that is, the modelling steps. The idea of providing a set of modelling steps as part of the proposed approach itself was inspired by [51]. It is very important to provide recommended modelling steps for any new modelling approach so as to maintain the same semantics in the model. In [51] modelling steps are proposed for class and state chart diagrams to represent Aspects. In a similar vein, the researcher attempts to provide a set of recommended modelling steps that can be followed when using the proposed approach. The contribution of this research lies in the fact that, in contrast to [51], the proposed AMM aims to provide modelling steps for all, not just a few, Aspectual UML diagrams. The 16 proposed AMM steps describe the activities and tasks that need to be carried out during the Aspectual UML modelling process.  Table 4 shows the steps in detail. It gives the order of the steps and the expected outputs (artefacts) of each of the steps. The outputs from preceding steps are then input to the subsequent steps.

Often OO UML diagrams are seen as just a random “bag of tools.” There is no preset order to their use; the same is applicable for AO. In other words, developers only draw the diagrams they need for their specific application, and only when they need them. However, the design process could be made easier if developers were able to refer to a comprehensive set of steps.

### 5.2. Aspectual UML Modelling Notation

**Figure 4:**
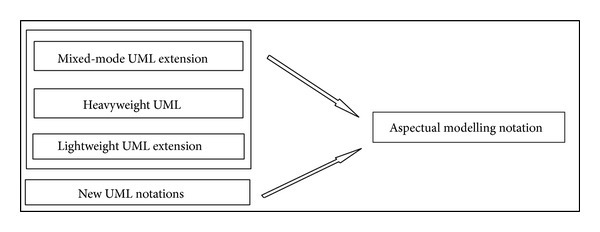
Sources for the proposed Aspectual UML modelling notation.

The majority of existing approaches focus on the abilities of UML extensions to model Aspects in AOSD, which basically depends on using stereotypes, tagged values, and constraints (i.e., a lightweight UML extension). However, there are no set standards for extension modelling that have yet been agreed on by the AOM community [21, 37]. Therefore this section proposes a comprehensive set of recommended Aspectual UML design modelling notations for AOSD. The researcher illustrates, shows, models, visualizes, and documents each Aspectual UML modelling view based on the proposed Aspectual UML modelling approach. The approach employs two UML extension abilities: the UML profile extension and the UML metamodel extension. In other words, the proposed Aspectual UML modelling approach is based on mixed-mode UML modelling as shown in Figure 4.

#### 5.2.1. Aspectual Structure Diagrams

**Figure 5:**
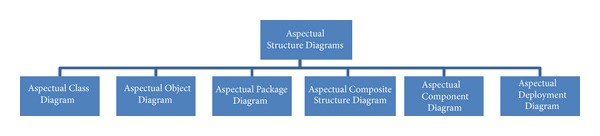
Aspectual structure diagrams.

Structure diagrams show the structure of a system and its parts on different abstraction and implementation levels and how they are related to each other. The elements in a structure diagram represent the meaningful concepts of a system, which may include abstract, real-world, and implementation concepts.  Figure 5 shows the Aspectual structure diagrams that were used to represent crosscutting concerns (Aspects) in the proposed comprehensive modelling approach.

The proposed approach provides an extension for each of the above six structural diagrams. Here, the researcher explains the Aspectual Class Diagram (ACD) extension. A Class Diagram provides a structural view of the system. Currently, a Class Diagram is unable to model Aspects and their crosscutting nature. This is the motivation for the proposed ACD, which can capture the static view of the system in terms of both Aspects and Classes, hence its name.  Table 5 illustrates the AO notations and their constructs, which can be used to support AspectJ in the context of the Class Diagram (structural view). Some of the notations to model Aspects using the Class Diagram are proposed by [34] and hence part of the ACD is inspired by their work.

**Figure 6:**
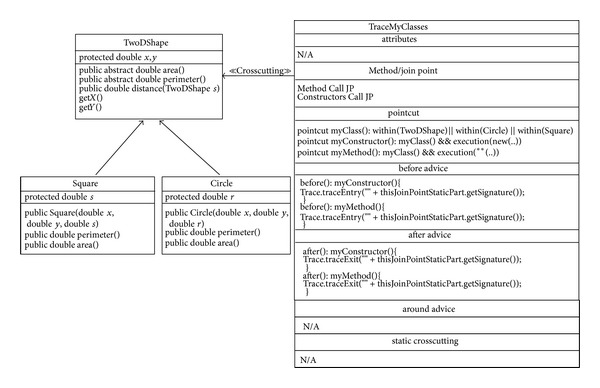
Aspectual Class Diagram for the Tracing Aspect.

The Aspect notation consists of the following four main sections: (i) the join point section indicates the join point signature and type; (ii) the pointcut section indicates the pointcut signature; (iii) the advice section shows the code that has to be injected at the pointcut; and (iv) the Aspect static crosscutting section illustrates the static crosscutting. To illustrate, Figure 6 shows the ACD of a Tracing Aspect.

From Figure 6, it can be seen that there are two Classes,  Square and Circle, which are inherited from the  TwoDShape Class. There is a crosscutting relation with an Aspect called  TraceMyclasses. The  TraceMyclasses  Aspect records all the methods and constructor calls by saving their signatures. Thus, the  TraceMyclasses  Aspect shows the join points and the pointcut designators that capture the join points such as

 
pointcut myClass(): within (TwoDShape) ||
 
within (Circle) || within (Square).

TraceMyclasses also shows the code to be ejected/executed before and after a specific join point such as

 
before (): myConstructor(){
 
Trace.traceEntry(""+
 
thisJoinPointStaticPart.get
 
Signature());
 

}

This Aspectual UML approach proposed a modelling notation to represent crosscutting concerns to support AspectJ for the rest of the UML structural diagrams.

**Figure 7:**
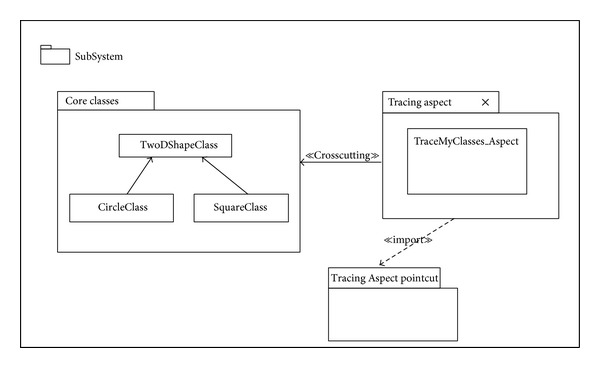
Aspectual Package Diagram for Tracing Aspect.

Another example for the Aspectual structural diagram is the Aspectual Package Diagram. A Package Diagram is a UML construct that enables the SA to organize model elements into groups, thereby making the UML diagrams simpler and easier to understand. It is also considered to be a piece of the model, as every part of a model belongs to one Package Diagram [52]. All the elements that belong to one package have to have common functionalities, viewpoints, and tightly related implementation. The APD in the proposed approach represents the Aspect and that Aspect's constructs.  Table 6 shows the proposed APD notations. Part of this APD has been adapted from [47], which proposes a package labelled with the Aspect stereotype on top, and some of the following Aspectual package relations have been inspired by [53].  Figure 7 shows the APD of the Tracing Aspect.

The third example of the Aspectual structural diagrams is the Aspectual Composite Structural Diagram (ACSD). Composite Structure Diagrams are used to explore run-time instances of interconnected instances collaborating over communications links to meet some common tasks. These diagrams are usually used to show the internal structure of a classifier and the classifier interaction with the environments through ports. In the proposed approach, this kind of diagram is called an ACSD because some new Aspectual notations have been added.  Table 7 describes the proposed Aspectual notations for the ASCD.

#### 5.2.2. Aspectual Behavioural Diagrams

**Figure 8:**
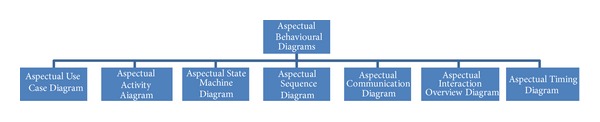
Aspectual behavioural diagrams.

Behavioural diagrams present the dynamic Aspects of the functionality of a system, but existing UML behaviour diagrams are unable to represent crosscutting concerns and their constructs.  Figure 8 shows the Aspectual behaviour diagrams that were considered in the proposed comprehensive approach. The researcher proposes some new extensions of the notations for these diagrams to represent crosscutting.

The Aspectual approach proposes an extension for the above six structural diagrams. Here, the researcher explains the Aspectual Sequence Diagram (ASD) as an example. A Sequence Diagram is one of the most important interaction diagrams. It mainly focuses on the messages (interactions) between Classes in terms of lifelines. The main concern of this type of diagram is the exchange of messages along with their occurrence in the lifeline of a specific Class. Currently, this diagram is incapable of representing crosscutting concerns. The proposed Aspectual modelling approach has enhanced the current design of the Sequence Diagram to capture Aspects.  Table 8 shows the modelling notations that have been added to create the ASD. Part of this work has been inspired by [54].

**Figure 9:**
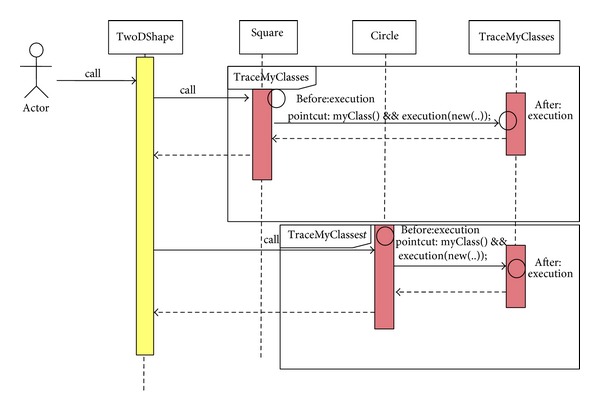
Aspectual Sequence Diagram for a Tracing Aspect.

As an example, Figure 9 gives the ASD for a Tracing Aspect. It shows how the Aspectual sequential interactions take place. There is one Aspect called  TraceMyclasses. The circle represents the advice location and type. If there is a piece of code (advice) to be injected before the join point then the  Before Advice  circle is provided, whereas if there is a piece of code (advice) to be injected after the join point then the  After Advice  circle is added.

This Aspectual UML approach proposed a modelling notation to represent crosscutting concerns to support AspectJ for the rest of the UML behavioural diagrams.

Another example of the Aspectual behavioural diagrams is the Aspectual Use Case Diagram. A Use Case Diagram is one of the behaviour diagrams in UML used to express a set of actions and tasks, that is, so-called use cases. The Use Case Diagram expresses the system by illustrating the collaboration between all use cases with one or more systems users (actors). The existing Use Case Diagram is not capable of representing the Aspects as nothing regarding Aspect representation is stated in the UML infrastructure and UML superstructure [55]. In the proposed approach, which aims to bridge this gap, the Use Case Diagram is developed to represent Aspects and is therefore called the AUCD.  Table 9 shows all the new Aspect notations and relations which are proposed to express the crosscutting nature of Aspects in the context of use cases. In developing the notations for the AUCD, the researcher makes use of the Aspect modelling notation proposed by [29].

**Figure 10:**
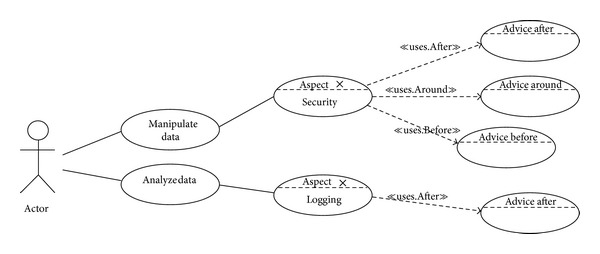
Example of Aspectual Use Case Diagram for Security Aspect.

Figure 10 illustrates the AUCD of the Security Aspect. It shows two main use cases, namely,   Manipulate Data  and  Analyse Data. Manipulate Data  is entangled with the  Security Aspect  at a join point where some piece of code has to be inserted to maintain security while the data is manipulated. So  Security Aspect  is executed by selecting the proper Aspectual use case, which might be  Before, After, or Around depending on the context.

The third example of the aspectual behavioural diagrams is the Aspectual Interaction Overview Diagram (AIOD). An Interaction Overview Diagram provides a general idea of the flow of control, where the nodes of the flow are interactions or interaction uses. It is different from the AD because the nodes are the interactions or interaction use. The Interaction Overview Diagram describes the interactions where some messages and lifelines might be hidden. It links a few diagrams that have already been drawn to represent a specific functionality. In its current form, the Interaction Overview Diagram cannot be utilized to represent Aspects [55]. To address this issue, the proposed Aspectual modelling approach includes new Aspectual modelling notations to represent Aspects in an AIOD.  Table 10 represents all the new modelling notations.

**Figure 11:**
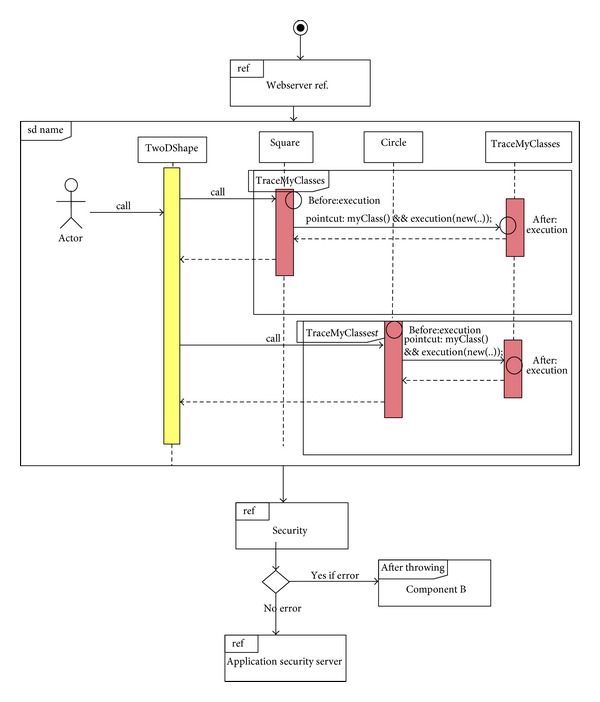
Aspectual Interaction Overview Diagram for Security and Traceability Aspects.

As an example, Figure 11 illustrates the AIOD for the  Security  and the  Traceability  Aspects. The AIOD shows how the two Aspects interact in the context of the interaction overview.

### 5.3. Aspectual UML Modelling Tool (AUMT) Based on UMLet 12.0

**Figure 12:**
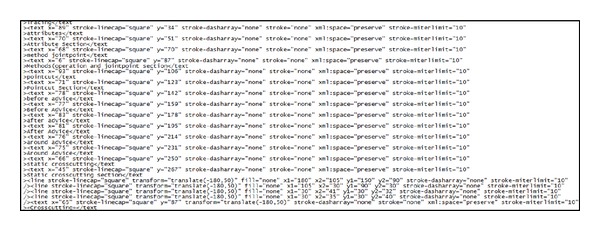
Example of SVG XML file generated by AUML tool.

**Figure 13:**
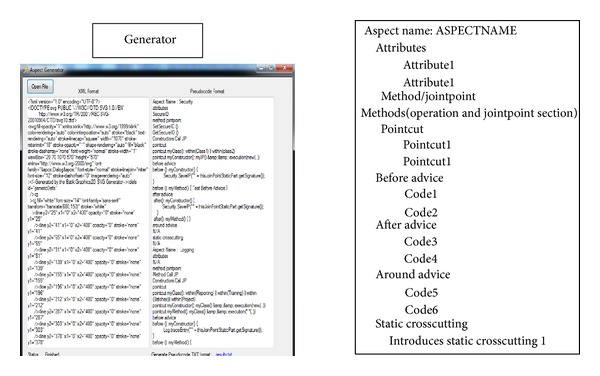
Example of AspectJ pseudocode generator.

**Figure 14:**
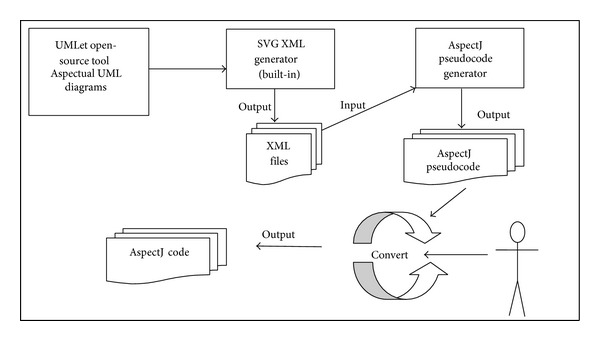
Process to generate AspectJ pseudocode.

There are two main reasons for developing the AUML tool: (1) the majority of the existing approaches do not develop their own tool to prove their proposed method and (2) the tool is used in this study to prove the applicability of the proposed theoretical approach. There are some interesting features of this tool, which help in enhancing the contribution of this research: (1) the AUML helps in generating XML SVG files out of the Aspectual drawings; (2) the AUML implements the semantic rules between the new proposed Aspectual notations and the existing OO notations and relations to maintain correct semantics and to not break the UML OMG standards; (3) the proposed AUML tool is able to generate AspectJ pseudocode from the UML drawings. These add-ins are added and integrated in UMLet. This pseudocode generator is built using the VB.NET 2010 framework. The UMLet tool generates the SVG XML files using the built-in XML generator as illustrated in Figure 12. After that, the XML file is used as an input to the AspectJ pseudocode generator, which is a parser as shown in Figure 13. This means that it searches and parses the XML files for special kinds of tags to generate a pseudocode; the generator gives the programmer the option to extract and copy the pseudocode. It also has the option to generate it in TXT file format. The software programmer then needs to work on the pseudocode generated to convert it to real code. This process is illustrated in Figure 14.

The proposed AUMT is a standalone tool for modelling and representing crosscutting concerns (Aspects) and their crosscutting nature using all the proposed Aspectual UML diagrams. The proposed tool supports AspectJ and provides a standalone environment for software designers which supports them in capturing, modelling, documenting, and representing Aspects and their constructs to produce correct software design and analysis artefacts, which are inputs in the later software development stages. This is crucial because, without such a tool, the erroneous representation of crosscutting concerns will result in inaccurate output, which will eventually lead to a lot of challenges in the later stages of development.

**Figure 15:**
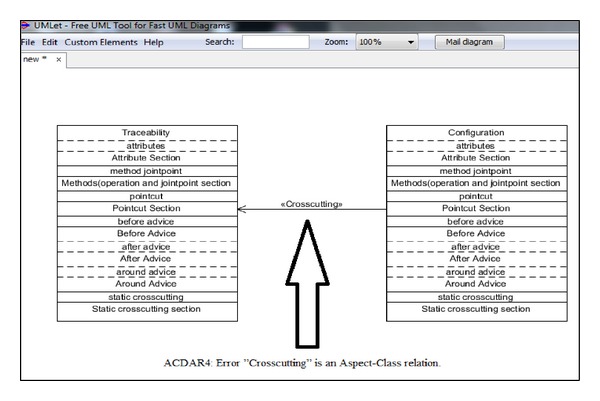
Proposed semantic rules for the proposed approach.

AUML defined semantic rules as the enforcement of the checking of all the Aspectual relations and notations to ensure that these Aspectual notations match the semantics of Aspect Orientation. These rules are derived from the semantic rules of the OMG UML 4.1 standard [55]. The Aspectual UML approach proposes new Aspectual UML notations and relationships, so it is critical to identify and understand the effects of the semantic changes of these new notations and relationships on the existing UML notations in order to maintain the semantic correctness of both Object-Oriented (OO) UML and Aspect-Oriented (AO) UML. For instance, the researcher proposes a new relation called “crosscutting.” The semantic rule specifies that this relation can only be drawn between one Aspect and one Class. In other words, if the designer attempts to draw this relation between two Classes or between two Aspects, then the proposed Aspectual UML modelling approach will not allow this based on the semantic rules proposed for this approach, as shown in Figure 15.

We have proposed some semantic rules/relationships for all Aspectual diagrams (behavioural and structural); these relocations/rules are there to maintain consistency and to make sure that OO semantic correctness is maintained.  Table 11 shows a sample of these AO semantic rules for Aspectual Class Diagram.

## 6. Experimental Results and Evaluation

Evaluation is a process of collecting and assessing the evidence against specific criteria for functionality and assurance. It can result in a measure of trust that indicates how well a system meets particular criteria [56]. This section presents the qualitative evaluation methods used to assess the proposed approach. The overall aims of this evaluation process are (1) to test that the proposed approach has appropriate functionality, (2) to address whether the proposed Aspectual UML approach offers a better means to handle Aspect modelling compared to the other AOM approaches, and (3) to confirm that the proposed Aspectual UML approach provides a better means for modelling crosscutting concerns. Various studies (e.g., [57, 58]) were reviewed to identify which evaluation methods would be best suited to perform this assessment. As a result, it was decided to use the following qualitative evaluation methods: (1) a focus group-based evaluation [59], (2) a “good design” criteria-based evaluation (the researcher consulted a LinkedIn group for researchers working in the field of Aspect Orientation called the “Aspect-Oriented Software Development Group,” which advised using this method along with other evaluation methods. Furthermore, [60] defined these “good design” criteria), and (3) an AspectJ support-based evaluation (it is a kind of think-aloud method proposed by Prof. Omar Aldawud (AOM expert)). Here, the researcher presents only general steps of the focus group evaluation method conduction.

### 6.1. Focus Group-Based Evaluation

**Figure 16:**
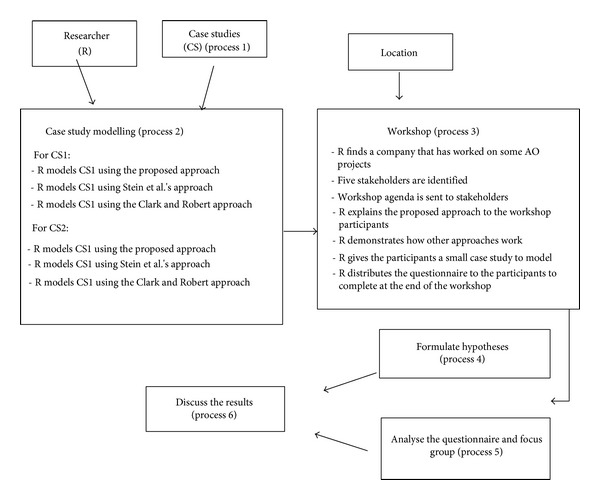
Focus group process and steps.

In this study, the goals of the focus group evaluation are to (1) investigate the applicability and validity of the proposed Aspectual UML approach and (2) draw on the expertise of the focus group to identify any omissions in the approach. There are two main reasons for selecting a focus-based method of evaluation for this research. First, a focus group can provide much more detailed information than other available methods. Second, some other evaluation methods such as the survey would not result in a significantly useful evaluation because the Aspect Orientation concept is not well known in the industry. Therefore, the researcher would not be able to find respondents with a good level of knowledge of the Aspect Orientation concept. The process of conducting the focus group evaluation is shown in Figure 16. The researcher faced a lot of challenge to find the right knowledgeable respondents; finally ten respondents were found.

To summarise these processes the author explains the proposed Aspectual UML approach to the participants; the different kinds of Aspects are explained and various topics are covered such as the reasons for developing a new approach, the types of new notations created and how they work, what improvements have been made, and the purpose of the Aspectual Modelling Methodology. The author as well explains how the other two selected approaches work, explains the two selected case studies and how they were modelled using the three approaches, shows the participants the drawings of each case study generated by each approach, gives the participants a small case study to try to model using the proposed Aspectual UML approach and gives them the option to try the proposed Aspectual Universal Modelling Language (AUML) tool as well, and lastly distributes a small questionnaire designed based on six hypotheses proposed and asks the participants to complete it and return it back by the end of the session.

#### 6.1.1. Process 1: Selecting the Case Studies

There are many case studies that could be used, but for this work the following two case studies are selected: (1) the European Ornithological Trust Survey System (EOTSS) and (2) the SCICOM Contact Centre Learning and Development Management System (SCCLDMS). The selection criteria used to choose the two case studies are as follows.

- *Size and Complexity.* These are significant criteria to consider when selecting case studies for use as part of an evaluation method. The two selected case studies are middle-sized and they are not complicated, which enables the researcher to present them comprehensively to the focus group during the workshop.
- *Quality.* Case studies must be of good quality and nontrivial in nature. These case studies have not been used as an example in the context of Aspect Orientation. Both of the selected case studies meet the quality requirement.
- *Real Project.* Case studies have to be real projects. The EOTSS was designed by the researcher as part of the researcher's Master's degree while the SCCLDMS was designed by the researcher for the researcher's previous employer.
- *Familiarity.* Case studies have to be familiar to the researcher so that the researcher can analyse them critically and then design and model them appropriately by using the proposed Aspectual UML approach. These two case studies meet this criterion because the researcher was directly involved in them.

*(a) Case Study 1: European Ornithological Trust Survey System (UOTSS).* The EOTSS is a real project that was undertaken by a nonprofit organization. The requirement text was obtained and analysed by the researcher to determine and model the concerns and the crosscutting concerns (Aspects). The EOTSS was used to set up a Digital Atlas of Breeding Birds (DABB). The DABB caters for the needs of environmental and wildlife preservation organizations and committees throughout Europe. The core functionalities of the EOTSS are to (1) record observations, (2) analyse observed data, (3) transfer data to a central collation system, and (4) transfer the configured data from the central system to the end users via various media.

*(b) Case Study 2: SCICOM Contact Center Learning and Development Management System.* The SCCLDMS is a real project that was developed by the researcher for a previous employer, SCICOM, as part of the NOKIA Contact Center Project. The specification was obtained from the company's training managers, human resource managers, and on-floor operation managers. The SCCLDMS includes the necessary features to meet the objectives of an organization such as keeping records, analysing data, and preparing reports to aid effective planning and decision making, which all are carried out online. This case study is chosen for two main reasons: (1) the researcher is quite familiar with the nature of the project and the environment which facilitates better and more accurate case study modelling and (2) it is a middle-sized case study.

#### 6.1.2. Process 2: Modelling the Case Studies

In this process, the researcher models the case studies by using the proposed Aspectual UML modelling approach and by using two other selected approaches. There are several approaches that could be used to model Aspects. In fact, as mentioned in SLR, there are 14 established Aspect-Oriented (AO) UML modelling approaches. However, due to research constraints, it is not possible to compare the proposed Aspectual UML approach against all of them, so just two of them are compared with the proposed approach. The researcher uses the two most effective approaches, the Composite Pattern approach [61] and the UML-based AO Design Notation for AspectJ approach [29].

- For evaluation of the proposed approach against heavyweight AODM using UML, the Composite Pattern approach of [61] is selected as a representative example because it is a well-known approach for heavyweight AODM [62]. In brief, the Composite Pattern approach makes use of some Subject-Oriented concepts and it consists of some steps to model the Aspects.
- For evaluation of the proposed approach against a lightweight AODM using UML, the UML-based AO Design Notation for AspectJ approach proposed by [14] is selected as a representative example because it has been implemented with the tool to support AspectJ and it is considered to be a well-known UML lightweight extension [62].

The above two approaches are accompanied by a lot of documentation, which helps the researcher to understand how exactly these two approaches work.

#### 6.1.3. Process 3: Conducting the Focus Group Workshop

**Figure 17:**
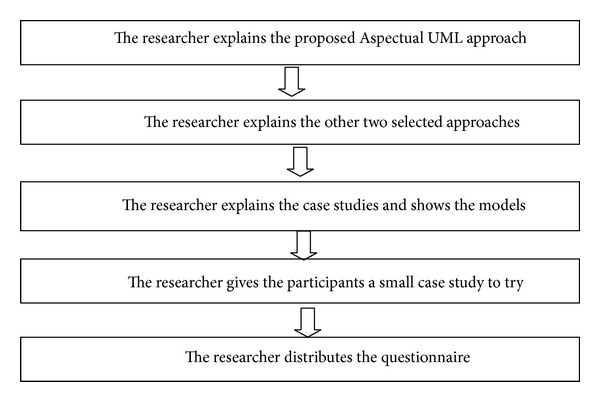
Workshop processes.

In this section earlier, we highlighted the difficulties of conducting an evaluation in this research field and explained the reasoning for the researcher's decision to conduct a workshop for a ten-person focus group consisting of industry professionals who have been involved in some Aspect Orientation projects.  Figure 17 shows the workshop conduction general process. In this workshop, the researcher

- explains the proposed Aspectual UML approach to the participants: the different kinds of Aspects are explained and various topics are covered such as the reasons for developing a new approach, the types of new notations created and how they work, what improvements have been made, and the purpose of the Aspectual Modelling Methodology;
- explains how the other two selected approaches work;
- explains the two selected case studies and how they were modelled using the three approaches and shows the participants the drawings of each case study generated by each approach;
- gives the participants a small case study to try to model using the proposed Aspectual UML approach and gives them the option to try the proposed Aspectual Universal Modelling Language (AUML) tool as well;
- distributes the questionnaire and asks the participants to complete it and return it back by the end of the workshop session.

The last step (distribute the questionnaire) is one of the most important steps of the focus group workshop. The questionnaire has been designed carefully to help answer the six hypotheses (formulated in Process 4), answer the research questions, and achieve the objectives of the thesis.

#### 6.1.4. Process 4: Formulating the Hypotheses

**Figure 18:**
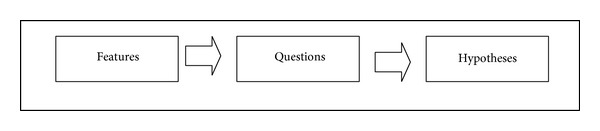
Hypotheses formulation.

In order for the researcher to formulate accurate hypotheses, the researcher has to list all Aspectual UML approach features, map them to questions, and then convert the questions into hypotheses, as shown in Figure 18.

Table 12 summarizes the mapping between the features, questions, and hypotheses.

#### 6.1.5. Processes 5 and 6: Analysing the Focus Group and Result Discussion

**Figure 19:**
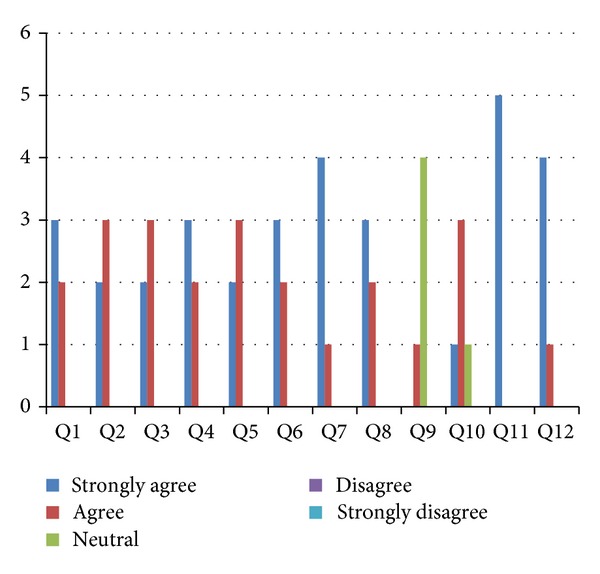
Scoring for all questions.

Six hypotheses were formulated in this research. To validate these hypotheses, a questionnaire was distributed to the participants by the end of the focus group. The results are shown in Figure 19.

In the final process of the focus group-based evaluation, the participants' comments, answers, decisions, and ratings collected in Process 5 were analysed to determine whether each of the six hypotheses identified in Process 3 could be confirmed, inconclusive, or unconfirmed.  Table 13 shows which question(s) were used to determine the result for each of the hypotheses.

(H1) The proposed approach is more comprehensive than the other AO UML modelling approaches.

The analysis of the participants' responses to Q1, Q6, Q7, and Q8, which are presented in Table 13 and Figure 19, confirms (H1).

(H2) The proposed approach provides a better means of representing Aspects based on AspectJ than the other AO approaches.

The analysis of the participants' responses to Q11 and Q12, which are presented in Table 13 and Figure 19, confirms (H2).

(H3) The proposed approach helps in increasing the consistency and maintainability between software development stages compared to the other AO approaches.

The analysis of the participants' responses to Q2, which are presented in Table 13 and Figure 19, confirms (H3).

(H4) The proposed approach provides a better means of modelling Aspects through the use of the proposed Aspectual modelling steps.

The analysis of the participants' responses to Q3 and Q10, which are presented in Table 13 and Figure 19, confirms (H4).

(H5) The proposed approach provides Aspectual UML design modelling notations that offer a better means of capturing and representing crosscutting concerns.

The analysis of the participants' responses to Q4, which are presented in Table 13 and Figure 19, confirms (H5).

(H6) The proposed approach provides Aspectual semantic rules that offer a better means of representing the semantics of the crosscutting concerns compared to the other AO approaches.

The analysis of the participants' responses to Q5, which are presented in Table 13 and Figure 19, confirms (H6).

Table 14 summarizes the above results.

### 6.2. AspectJ Support-Based Mapping

Part of the evaluation methods used to assess the proposed approach is an AspectJ support-based mapping. Due to the claim that the proposed approach supports AspectJ, the researcher has to conduct the mapping. In this mapping task, the researcher tries to compare the proposed modelling notations against the original AspectJ constructs. The researcher looks at all the detailed AspectJ constructs to see how they would be represented and mapped in the proposed modelling approach for different kinds of UML diagram. This evaluation method should endorse the overall evaluation of the proposed approach if the value given is “met” for all AspectJ detailed constructs. The researcher used the think-aloud evaluation technique in this evaluation as well [63].

For the purpose of this mapping, the researcher assesses the Aspectual Class Diagram (ACD), which was explained in the previous sections. The AspectJ constructs that the researcher compares the ACD against are provided in [64]. In AspectJ, as in any programming language, many details of the programming constructs can only appear at the programming level. Thus, even though the proposed approach is designed and developed to support AspectJ, it shows abstract views of the system with small details. In other words, the proposed approach models the system based on AspectJ's detailed constructs, but it does not concern itself with every detailed programming construct. For instance, AspectA and AspectB inject codes at the same join point P1, and there is a precedence/priority setting to determine which of the two Aspects injects first and which comes second. This process is modelled by the proposed approach using “precedence” relationships. However, the researcher does not pay attention to how precedence itself takes place in reality because this involves very detailed programming which is not the issue of interest in this thesis.

#### 6.2.1. Join Point of AspectJ

**Figure 20:**
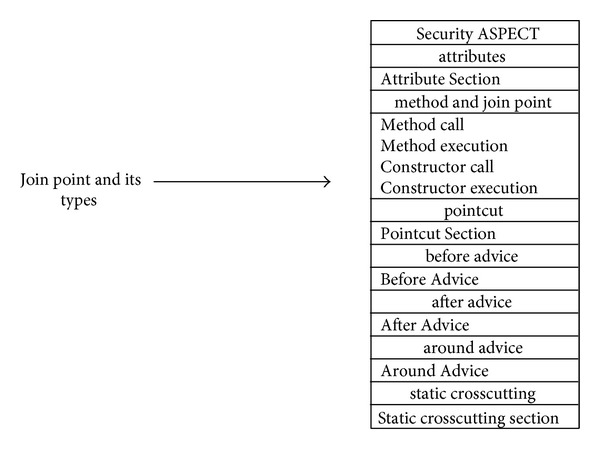
Join Point Types for Security Aspect.

A join point is a well-defined point in the execution of a program. There are many types of join point [64]. The proposed ACD can represent all the join points to model the join point signature and type, as shown in Figure 20.

Figure 20 illustrates an Aspect called Security. It does not show all the details of the Aspect but depicts the representation of the join points. From the figure, it can be seen that even though the Aspect defines the crosscutting behaviour generally, it also defines the types of join point that cut across the points in a program's execution. The proposed modelling shows these join points in the early stage of the design process. The benefit of showing the join points and their types early on is that the programmers will have a more complete picture of the nature of the join point that they will expose and the crosscutting effect that will take place, and this knowledge will make the development process easier for them.

The same goes for the other types of join point that were explained in [64]. The researcher can have them all in this join point section or just the needed ones. This is the case for the ACD. The proposed approach gives programmers the ability to model the join point and its types by using the other Aspectual UML behavioural and structural diagrams as well.

#### 6.2.2. Pointcut of AspectJ

**Figure 21:**
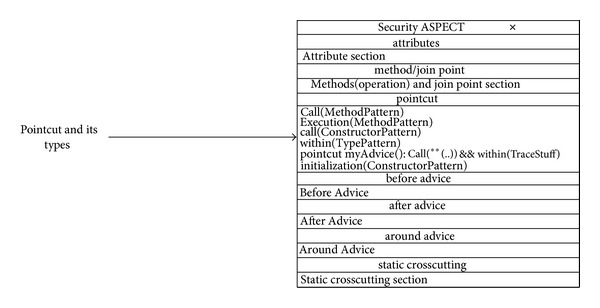
Pointcut types for Security Aspect.

A pointcut is a program element that picks out join points and exposes the data from the execution context of those join points [64]. Similar to join points, there are many types of pointcut, which were elaborated in [64]. The proposed ACD can represent all the pointcuts to model the pointcut signature and type, as shown in Figure 21.

Figure 21 also illustrates an Aspect called Security. It does not show all the details of the Aspect but depicts the representation of the pointcuts and information on the signature and type to some extent. From the figure, it can be seen that there are different types of pointcut in the pointcut section. Also, there is no fixed format and/or signature for the pointcut, so the researcher has provided a flexible pointcut section, which gives programmers the ability to model any pointcut that is needed. The benefit of modelling these pointcuts is almost the same as for the join points; that is, it can provide an overview of the effect of crosscutting concerns so the programmer knows which pointcuts need to be implemented and it makes the task of developing the software a bit easier. In particular, it helps the programmer in making the stages of system development maintainable and consistent.

The same goes for the other types of pointcut that were explained in [64]. The researcher can have them all in this pointcuts section or the needed ones, as the proposed representation provides a free-style writing/modelling tool. This is the case for ACD. The proposed approach gives programmers the ability to model the pointcut and its types by using the other Aspectual UML behavioural and structural diagrams as well.

It should be noted that the proposed approach does not pay attention to very detailed matters concerning pointcuts such as the accurate syntax of pointcut definitions, the pointcut signature, and the matching and type of patterns. In the context of this study, the researcher is interested in preserving the SoC and does not want to interfere with the concerns of the other stages of the software life cycle.

#### 6.2.3. Advice of AspectJ

**Figure 22:**
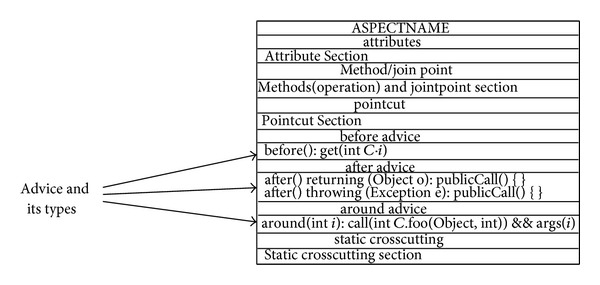
Advice modelling for a sample Aspect.

The advice construct defines crosscutting behaviour and this piece of code runs at every join point picked by a specific pointcut. Specifically, the code that runs depends on the type of advice. AspectJ defines three types of advice, which are modelled by the researcher. These types determine how the advice interacts with the join point: “After” advice runs after the join point, “Before” advice runs before the join point, and “Around” advice runs to replace the join point. “After” advice is divided into two more types: After Returning and After Throwing [8]. The proposed approach provides a modelling notation for all these advice constructs.  Figure 22 illustrates these kinds of advice modelled in the ACD.

Figure 22 illustrates a sample Aspect. It does not show all the details of the Aspect but depicts the advice modelling applied in the ACD. Examples of the types of advice are shown in Figure 22 in the advice section. Also, there is no fixed format and/or signature for the advice, so the researcher has provided a flexible advice section, which gives programmers the ability to model any advice necessary. The benefits of modelling these types of advice are almost the same as for join points and pointcuts in that software developers can gain an overview of the effect of crosscutting concerns, so they know what types of main advice have to be implemented and this makes the developer's task a bit easier.

That was the case for the ACD. The proposed approach gives programmers the ability to model the advice and its types by using the other Aspectual UML behavioural and structural diagrams as well. Finally, it should be noted that the proposed approach does not pay attention to all the detailed matters concerning advice such as the accurate signature, modifiers, and precedence of the advice types because the researcher cares about preserving the SoC and does not want to interfere in the implementation stage.

#### 6.2.4. Aspect of AspectJ

**Figure 23:**
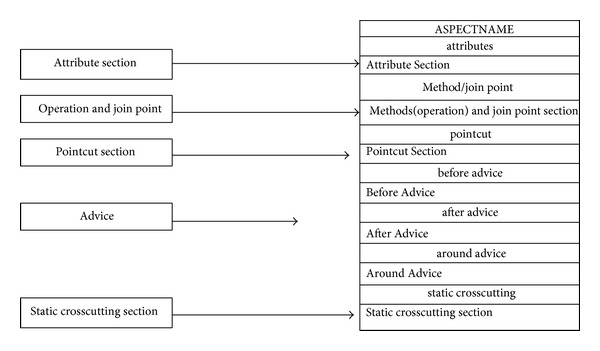
Representation of an Aspect in the Aspectual Class Diagram.

The Aspect is the element that contains and encapsulates all the constructs. However, this does not necessarily mean that all these constructs (join point, pointcut, advice, and introduction) appear in one Aspect. The content of the Aspect basically depends on the crosscutting nature of that Aspect. The proposed approach provides a modelling notation to represent Aspects. The Aspect notation in the ACD gives a name and a separate section to each one of the AspectJ constructs, where applicable.  Figure 23 illustrates the representation of an Aspect in the ACD.

Figure 23 illustrates how an Aspect is represented by using the notations of the ACD. From the figure, it can be seen that the Aspect represents all the other crosscutting constructs (advice, pointcut, etc.). However, it should be noted that the proposed Aspect representation does not address all the issues relating to the implementation of an Aspect, such as Aspect declaration, instantiation, and Aspect privilege as this would interfere with the implementation part of the system developer. The Aspect can be modelled by using different kinds of Aspectual UML diagrams.

## 7. Conclusion and Future Work

The researcher has presented a summary of the work undertaken in this study. The study contributes to the field by proposing a suitable UML extension for the design of AO systems to support AspectJ. The proposed approach is divided into two main parts. The first part concerns the Aspectual design model, which the researcher developed in order to be able to visualize, draw, and model crosscutting concerns. The second part is the AMM, which consists of some recommended steps that developers can follow when modelling crosscutting concerns using the proposed approach. Another contribution consists of the development of some Aspectual semantic rules to control the structure of the model, which the researcher has devised to ensure that the modelling of the Aspects to support AspectJ maintains and conveys meaningful and correct semantics when Aspects interact with each other as well as when they interact with the core Classes and other OO notations. Lastly, the researcher has also attempted to develop an approach and tool that can be used to generate AspectJ pseudocode.

To conclude, it is clear to the researcher that AODM has a very wide scope in terms of application and that there will always be new developments in this technology and room for researchers to innovate and add to existing knowledge. A number of suggestions regarding ways to improve the proposed approach are briefly outlined below.

- The proposed prototype could be developed as an online tool that could be utilized by various system analysts in different locations.
- The model could be further developed to support the design of smartphones and connected devices (e.g., tablets).
- The proposed modelling approach is to support a specific AOP language (AspectJ), so it might be worthwhile to investigate how to extend the proposed modelling approach to be language independent and to cover other AOP languages such as JAsCo, Spring, and Aspect Werkz.
- The tool could be improved by adding more functionality, for instance, to check semantic rules and apply them automatically.

## Figures and Tables

**Table 1:** Articles identified for the research study.

Research question	Total number of references retrieved	Number of studies after excluding duplicates	Number of abstracts screened	Number of studies after abstracts were screened	Number of full texts screened	Number of selected studies	Final number of relevant studies
RQ1	285	242	242	82	70	64	64
RQ2	160	132	102	55	7	6	6
RQ3	23	16	13	10	6	4	3

Total	468	390	357	147	83	74	73

**(a):** 

Approach	Language specification									
	UMLV	EM		LP	PD	DT		MP	T	A
		Metamodel	UML profile			Behavioural	Structural			
[17]	1.x	Y	N	G	Y	SD	CD/UPD	N	Y	P
[27]	2.0	N	N	G	N	N	CD	N	N	N
[22]	1.x	N	N	G	N	N	COD/CD	Y	Y	Y
[24]	1.1	Y	N	G	N	N	CD	T	T	Y
[26]	2.0	Y	N	G	Y	UC/SD/COMMD	CD/COD	N	Y	Y
[32]	2	N	Y	G	N	N	UPD	N	N	N
[35]	2.0	N	Y	G	N	SD	N	N	N	N
[34]	1.x	Y	Y	S	Y	N	CD	N	N	N
[25]	2.0	Y	N	G	N	N	CD/PD	N	N	N
[29]	1.x	Y	N	S	Y	N	CD/CLD	N	P	P
[30]	2.0	N	Y	G	N	SD/STD	CD/DD	N	Y	A
[31]	1.x	N	Y	G	N	STD	CD	P	P	N
[28]	2.2	Y	N	G	N	N	CD/PD	N	Y	Y
[33]	2.3	Y	N	S	Y	SD	CD	N	Y	Y

**(b):** 

Coding index					
UML version	UMLV	No	N	Partial traceability	PT
Extension mechanism	EM	Class Diagram	CD	State Diagram	STD
Language purpose	LP	Package Diagram	UPD	Yes	Y
Platform dependency	PD	Partial support	P	Adaptability	A
Diagram type	DT	Component Diagram	COD	Sequence Diagram	SD
Modelling process	MP	Use Case	UC	Specific/general	S/G
Traceability	T	Communication Diagram	COMMD	Collaboration Diagram	CLLD

**Table 3:** Summary of the conceptual framework.

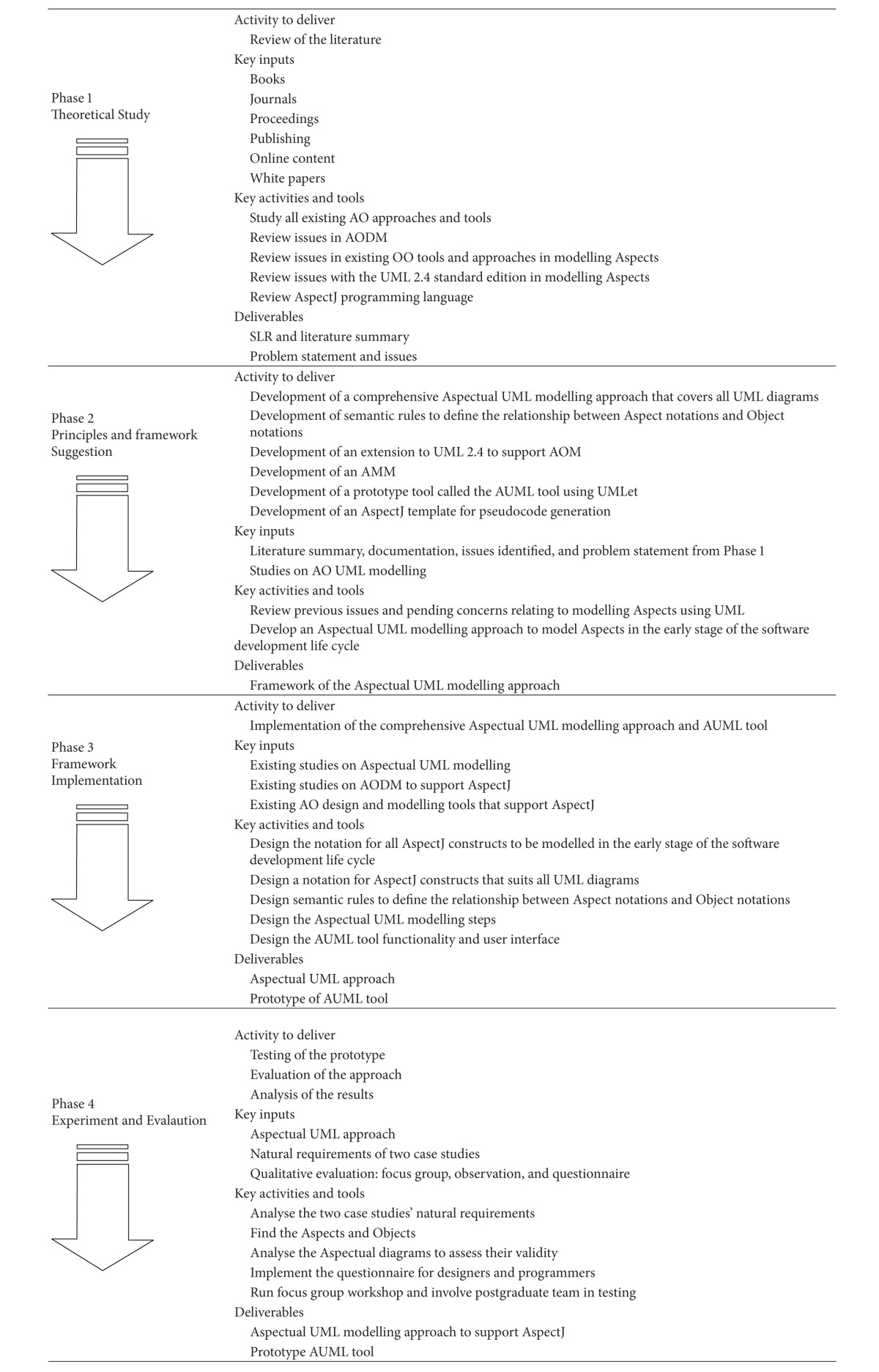

**Table 4:** AMM steps.

Number	Description	Expected output(s)
1	Analyse the natural requirement lists and identify the list of expected Objects (concerns)	List of Objects/concerns
2	Identify the list of expected system Aspects (crosscutting concerns)	List of Aspects/crosscutting concerns
3	Define the relations between the Classes themselves, Classes-Aspect and Aspect-Aspect relationships	Identification of relationships
4	Draw the Aspectual Use Case Diagram (AUCD)	Aspectual Use Case Model (use case view)
5	Draw the Aspectual Activity Diagram (AAD)	Aspectual Activity Model (activity view)
6	Draw the Aspectual Class Diagram (ACD)	Aspectual Class Model (static view)
7	Draw the Aspectual Object Diagram (AOD)	Aspectual Object Model (static view)
8	Draw the Aspectual State Machine Diagram (ASMD)	Aspectual State Machine Model (state machine view)
9	Draw the Aspectual Sequence Diagram (ASD)	Aspectual Sequence Model (interaction view)
10	Draw the Aspectual Communication Diagram (ACmD)	Aspectual Communication Model (interaction view)
11	Draw the Aspectual Timing Diagram (ATD)	Aspectual Timing Model (interaction view)
12	Draw the Aspectual Interaction Overview Diagram (AIOD)	Aspectual Interaction Overview Model (interaction view)
13	Draw the Aspectual Package Diagram (APD)	Aspectual Package Model (model management view)
14	Draw the Aspectual Composite Structure Diagram (ACSD)	Aspectual Composite Structure Model (interaction view)
15	Draw the Aspectual Component Diagram (ACoD)	Aspectual Component Model (physical/implementation view)
16	Draw the Aspectual Deployment Diagram (ADD)	Aspectual Deployment Model (physical/implementation view)

**Table 5:** Representation of an Aspect in the Aspectual Class Diagram.

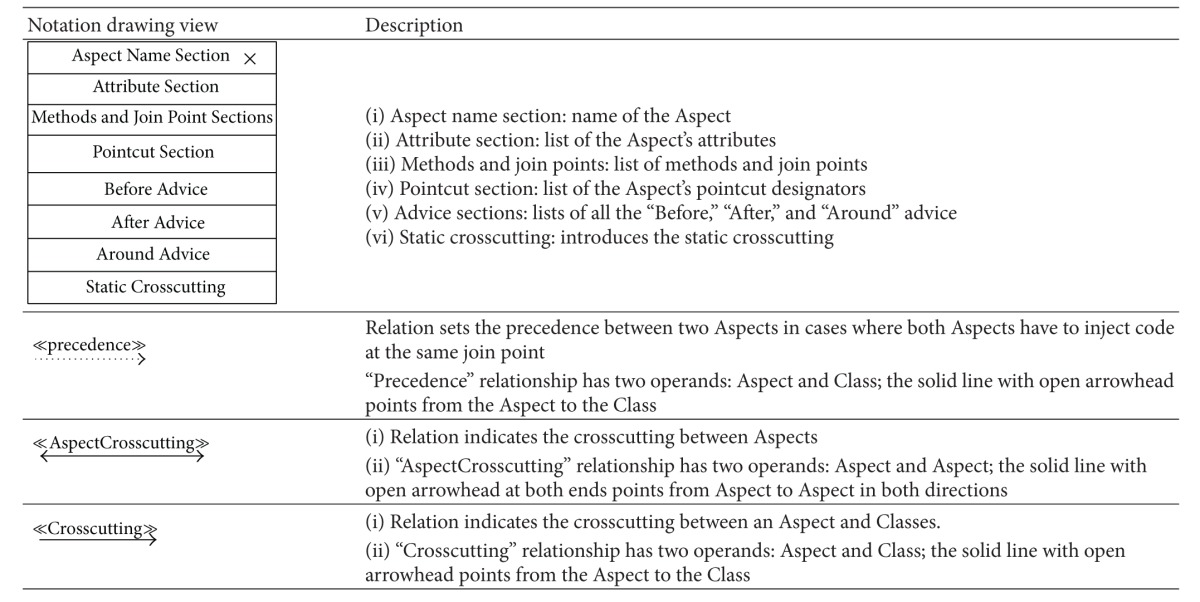

**Table 6:** Representation of an Aspect in the Aspectual Package Diagram.

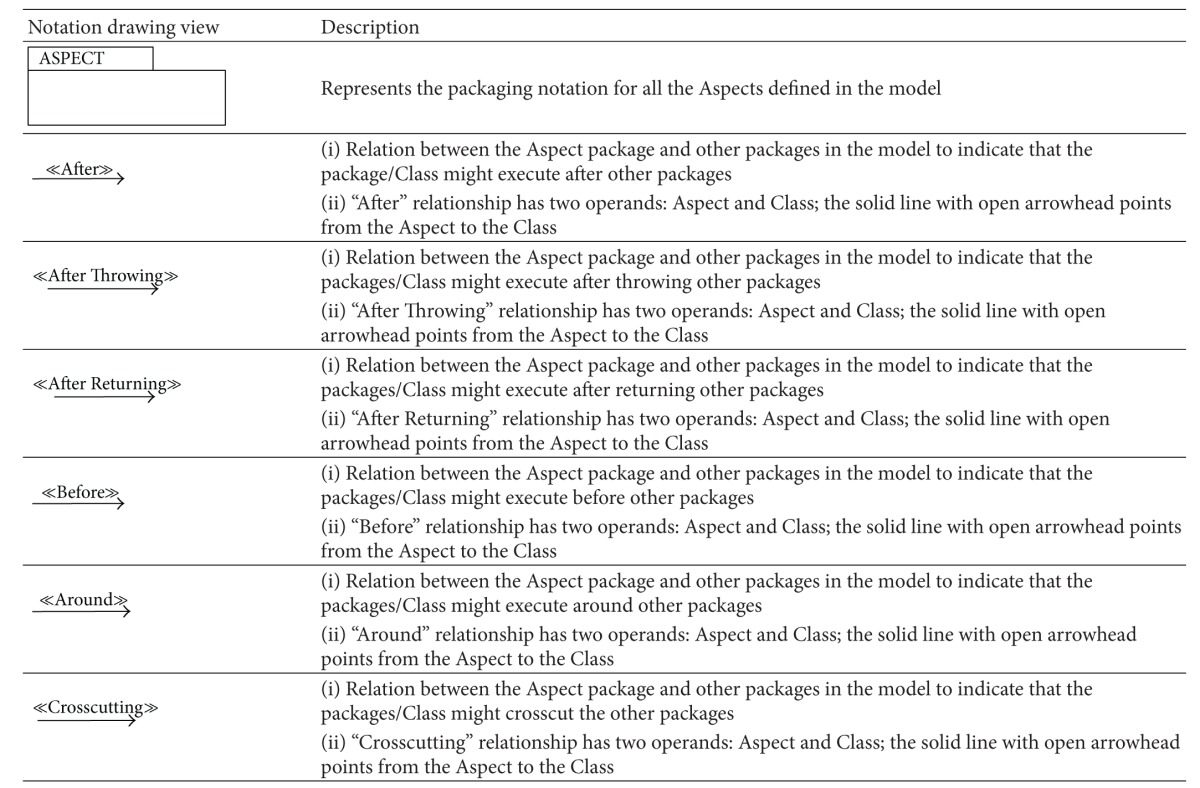

**Table 7:** Representation of an Aspect in the Aspectual Composite Structure Diagram.

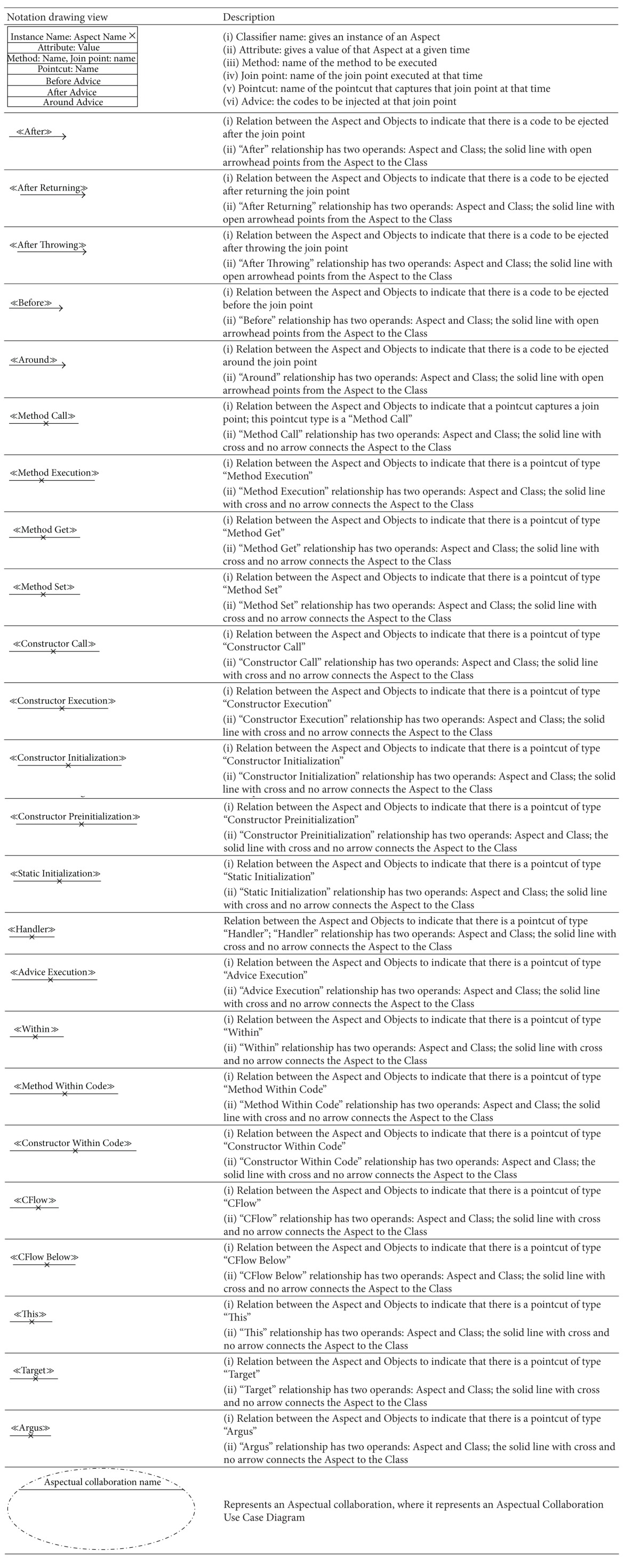

**Table 8:** Representation of an Aspect in the Aspectual Sequence Diagram.

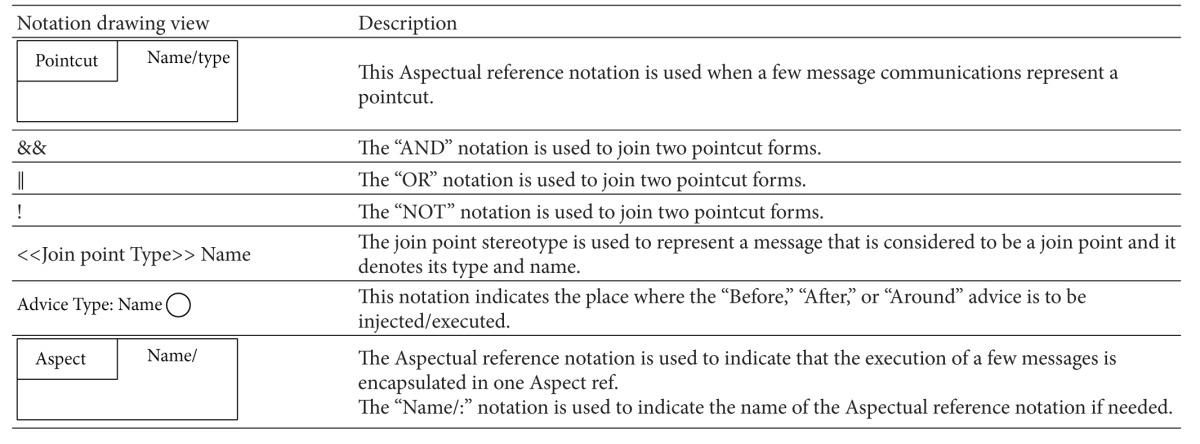

**Table 9:** Representation of an Aspect in the Aspectual Use Case Diagram.

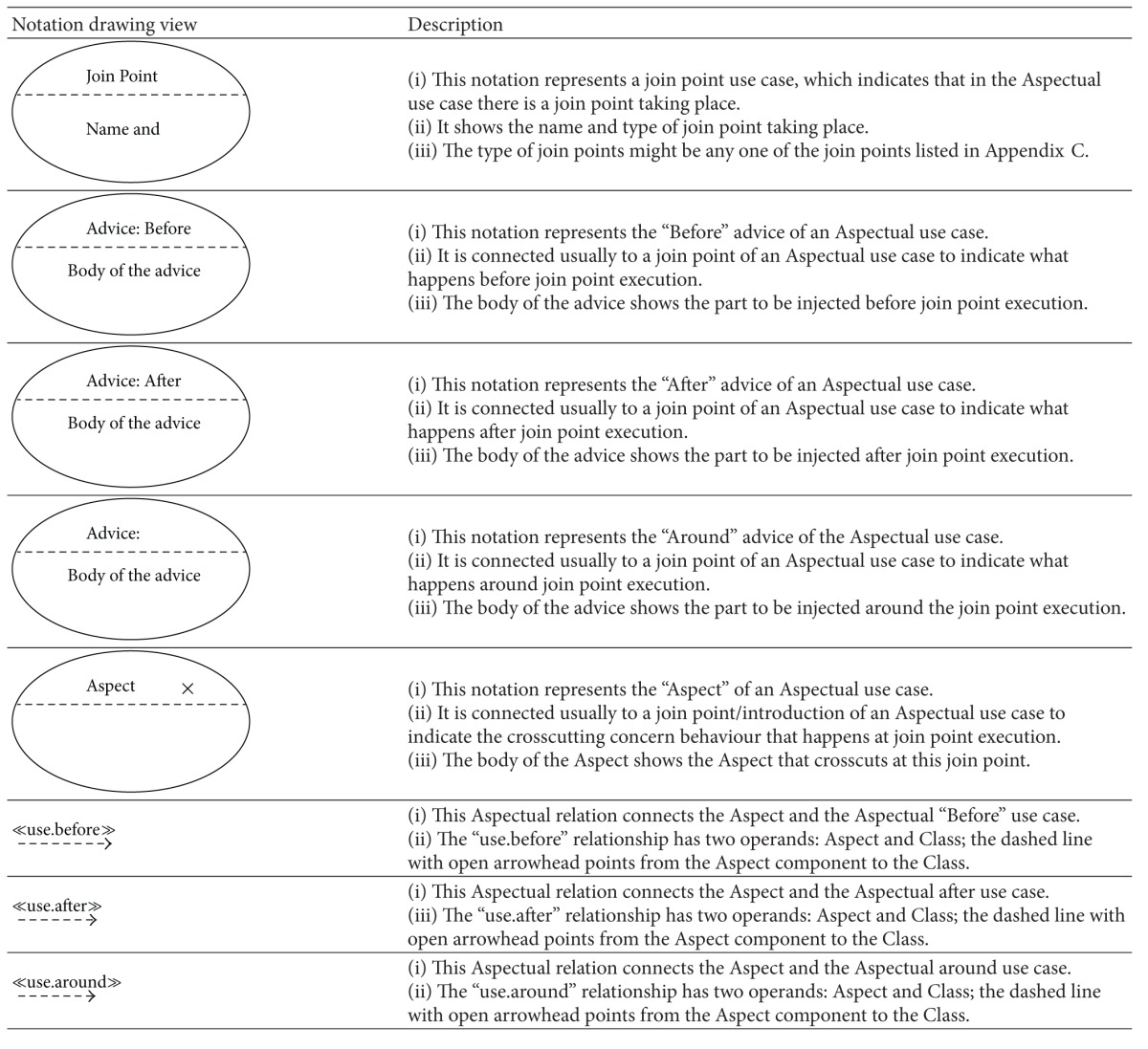

**Table 10:** Representation of an Aspect in the Aspectual Interaction Overview Diagram.

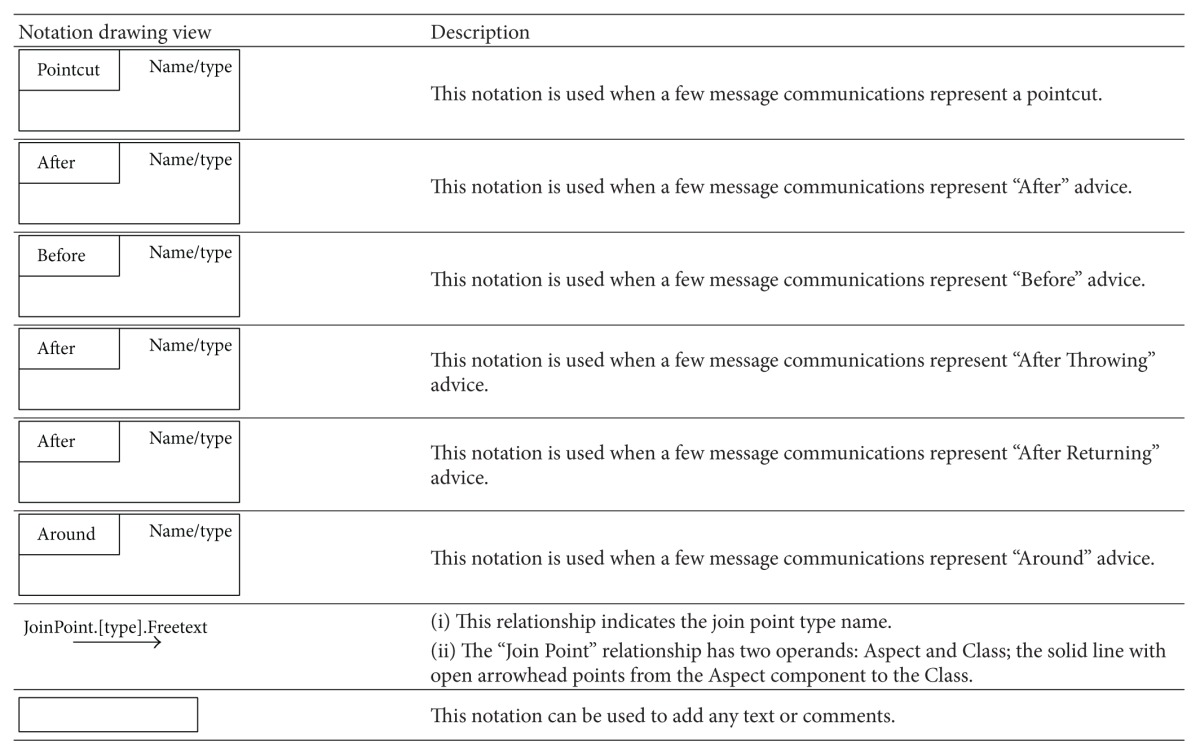

**Table 11:** Aspectual Class Diagram semantic rules.

Aspectual Class Diagram rules	
Semantic rule	Description
No Aspect name given	This semantic rule means that all Aspects should have a name.

“precedence” is an Aspect-Aspect relation	This semantic rule means that “precedence” is an Aspect-Aspect relationship. This means that the user (system analyst (SA)) should not attempt to use the “precedence” relationship with a Class at any end. The “precedence” relationship takes place only between Aspects, when both have to eject a piece of code at the same join point. So the “precedence” relation sets which of the two Aspects executes the code first.

“Aspect crosscutting” is an Aspect-Aspect relation	This semantic rule means that “Aspect crosscutting” is an Aspect-Aspect relationship. This means that the SA should not attempt to use the “Aspect crosscutting” relationship with a Class at any end. The “Aspect crosscutting” relationship takes place when two Aspects are cut across each other.

“crosscutting” is an Aspect-Class relation	This semantic rule means that “crosscutting” is an Aspect-Class relationship. This means that the SA should not attempt to use it with no Class at any end. The “crosscutting” relationship takes place between an Aspect and a Class to show that there is crosscutting.

This is an OO notation	This semantic rule means that this is an “OO notation.” This means that the SA should not attempt to use OO relations such as dependency and inheritance as a relation with an Aspect at any end. The OO notation cannot be connected to AO notations.

**Table 12:** Mapping of the features, questions, and hypotheses.

Feature	Question	Hypothesis
The proposed Aspectual approach is a comprehensive framework because it supports all UML structural and behavioural diagrams.	Is the proposed approach more comprehensive than the other AO approaches?	**H1:** The proposed approach is more comprehensive than the other AO UML modelling approaches.

The proposed Aspectual approach is implemented to support AspectJ's detailed constructs.	Does the proposed approach provide a better means of representing Aspects based on AspectJ than the other AO approaches?	**H2:** The proposed approach provides a better means of representing Aspects based on AspectJ than the other AO approaches.

The proposed Aspectual approach helps in increasing the consistency between software development stages.	Does the proposed approach help in increasing the consistency between software development stages compared to the other AO approaches?	**H3:** The proposed approach helps in increasing the consistency between software development stages compared to the other AO approaches.

The proposed approach provides Aspectual UML modelling steps.	Does the proposed approach provide a better means of modelling Aspects through the use of the proposed Aspectual UML modelling steps?	**H4:** The proposed approach provides a better means of modelling Aspects through the use of the proposed Aspectual UML modelling steps.

The proposed approach provides Aspectual UML design modelling notation to support AspectJ	Does the proposed approach provide Aspectual UML design modelling notations that offer a better means of capturing and representing crosscutting concerns?	**H5:** The proposed approach provides Aspectual UML design modelling notations that offer a better means of capturing and representing crosscutting concerns.

The proposed Aspectual UML approach provides Aspectual semantic rules to control the structure of the model.	Does the proposed approach provide Aspectual semantic rules that offer a better means of representing the semantics of the crosscutting concerns compared to the other AO approaches?	**H6:** The proposed approach provides Aspectual semantic rules that offer a better means of representing the semantics of the crosscutting concerns compared to the other AO approaches.

**Table 13:** Hypothesis and question matching.

Hypothesis	Question number
H1	Q1, Q6, Q7, Q8
H2	Q11, Q12
H3	Q2
H4	Q3, Q10
H5	Q4
H6	Q5

**Table 14:** Hypotheses results.

Hypothesis number	Hypothesis result
H1	Confirmed
H2	Confirmed
H3	Confirmed
H4	Confirmed
H5	Confirmed
H6	Confirmed
